# Interaction of Antibiotics with Innate Host Defense Factors against *Salmonella enterica* Serotype Newport

**DOI:** 10.1128/mSphere.00410-17

**Published:** 2017-12-06

**Authors:** George Sakoulas, Monika Kumaraswamy, Armin Kousha, Victor Nizet

**Affiliations:** a University of California San Diego School of Medicine, La Jolla, California, USA; b Skaggs School of Pharmacy, University of California San Diego, La Jolla, California, USA; Escola Paulista de Medicina/Universidade Federal de São Paulo

**Keywords:** cathelicidin, innate immunity, meningitis, *Salmonella*

## Abstract

This study examines the pharmacodynamics of antimicrobials that are used to treat *Salmonella* with each other and with key components of the innate immune system. Antimicrobial synergy was assessed using time-kill and checkerboard assays. Antimicrobial interactions with innate immunity were studied by employing cathelicidin LL-37, whole-blood, and neutrophil killing assays. Ceftriaxone and ciprofloxacin were found to be synergistic *in vitro* against *Salmonella enterica* serotype Newport. Ceftriaxone, ciprofloxacin, and azithromycin each demonstrated synergy with the human cathelicidin defense peptide LL-37 in killing *Salmonella*. Exposure of *Salmonella* to sub-MICs of ceftriaxone resulted in enhanced susceptibility to LL-37, whole blood, and neutrophil killing. The activity of antibiotics *in vivo* against *Salmonella* may be underestimated in bacteriologic media lacking components of innate immunity. The pharmacodynamic interactions of antibiotics used to treat *Salmonella* with each other and with components of innate immunity warrant further study in light of recent findings showing *in vivo* selection of antimicrobial resistance by single agents in this pathogen.

**IMPORTANCE** It is becoming increasingly understood that the current paradigms of *in vitro* antimicrobial susceptibility testing may have significant shortcomings in predicting activity *in vivo*. This study evaluated the activity of several antibiotics alone and in combination against clinical isolates of *Salmonella enterica* serotype Newport (meningitis case) utilizing both conventional and physiological media. In addition, the interactions of these antibiotics with components of the innate immune system were evaluated. Azithromycin, which has performed quite well clinically despite high MICs in conventional media, was shown to be more active in physiological media and to enhance innate immune system killing. Alternatively, chloramphenicol did not show enhanced immune system killing, paralleling its inferior clinical performance to other antibiotics that have been used to treat *Salmonella* meningitis. These findings are important additions to the building understanding of current *in vitro* antimicrobial assay limitations that hopefully will amount to future improvements in these assays to better predict clinical efficacy and activity *in vivo*.

## Abstract (precis)

It is becoming increasingly understood that the current paradigms of *in vitro* antimicrobial susceptibility testing may have significant shortcomings in predicting activity *in vivo*. This study evaluated the activity of several antibiotics alone and in combination against clinical isolates of *Salmonella enterica* serotype Newport (meningitis case) utilizing both conventional and physiological media. In addition, the interactions of these antibiotics with components of the innate immune system were evaluated. Azithromycin, which has performed quite well clinically despite high MICs in conventional media, was shown to be more active in physiological media and to enhance innate immune system killing. Alternatively, chloramphenicol did not show enhanced immune system killing, paralleling its inferior clinical performance to other antibiotics that have been used to treat *Salmonella* meningitis. These findings are important additions to the building understanding of current *in vitro* antimicrobial assay limitations that hopefully will amount to future improvements in these assays to better predict clinical efficacy and activity *in vivo*.

## INTRODUCTION

Salmonellosis is one of the most common causes of diarrhea worldwide. Global estimates are 90,000 deaths from nontyphoidal and 180,000 deaths from typhoidal salmonellosis, while annual estimates in the United States are that about 1.2 million cases and 450 deaths occur from nontyphoidal salmonellosis (1, 2).

*Salmonella* meningitis is an unusual complication of salmonellosis in developed countries, although it is an emerging threat in resource-poor parts of the world. Most cases described in the literature occur in very young children or otherwise immunocompromised adults and are most commonly caused by typhoidal strains (3–5). However, rare cases have been described in immunocompetent adults, including in one neurosurgical patient (6, 7). Outcomes are generally poor, and treatment is not standardized due to the small number of reported cases (8). Nontyphoidal *Salmonella* meningitis in immunocompetent adults is exceedingly rare, and as a result, therapy is even less defined. Given the very complex pharmacodynamic interactions between coadministered antibiotics and endogenous immune factors, including the potential selection of antimicrobial resistance (9), we examined how different antibiotics influence *Salmonella* susceptibility to killing by cathelicidin LL-37, a critical immune component in meningeal infection (10, 11), as well as whole blood and neutrophils.

## RESULTS

### Antimicrobial susceptibility and synergy assays.

Susceptibility testing was performed for cerebrospinal fluid (CSF) and blood *Salmonella* strains using cation-adjusted Mueller-Hinton broth (CA-MHB) and RPMI 1640 medium supplemented with 10% Luria-Bertani broth (RPMI+10% LB). MIC results for ceftriaxone (CRO), ciprofloxacin (CIP), azithromycin (AZM), chloramphenicol (CM), and cathelicidin LL-37 are shown in Table 1. The respective MICs in RPMI+10% LB and CA-MHB of CRO (0.063 versus 0.125 mg/liter), CIP (0.0125 versus 0.05 mg/liter), and AZM (4 versus >32 mg/liter) were consistently lower in the physiologic medium. No difference in MIC was observed for CM (0.5 mg/liter) between the two media.

**TABLE 1:** MIC of antimicrobials against *Salmonella* strains from CSF and blood

*Salmonella* sample	MIC in medium^a^								
	CRO (mg/liter)		CIP (mg/liter)		AZM (mg/liter)		CM (mg/liter)		LL-37 in R (μM)^b^
	M	R	M	R	M	R	M	R	
CSF	0.125	0.063	0.05	0.0125	>32	4	0.5	0.5	32
Blood	0.125	0.063	0.05	0.0125	>32	4	0.5	0.5	32

a CRO, ceftriaxone; CIP, ciprofloxacin; AZM, azithromycin; CM, chloramphenicol; M, cation-adjusted Mueller-Hinton broth; R, RPMI+10% LB.

b LL-37 testing was performed only in RPMI-LB, as it is not active in MHB.

Kill curves at 2×, 4×, and 8× MIC were performed for CRO, CIP, and CM in both CA-MHB and RPMI+10% LB and for AZM in RPMI+10% LB for the CSF *Salmonella* isolate. The results in Fig. S1 in the supplemental material demonstrate bactericidal activity for all drugs except CM, which was bacteriostatic.

10.1128/mSphere.00410-17.1 FIG S1  Kill curves against CSF *Salmonella enterica* strain in MHB (top row) and RPMI+10% LB (bottom row) using various antibiotics at 2×, 4×, and 8× MIC. Azithromycin was assayed only in RPMI+10% LB, as it was inactive in MHB. Download FIG S1, PDF file, 0.1 MB. Copyright © 2017 Sakoulas et al. 2017 Sakoulas et al. This content is distributed under the terms of the Creative Commons Attribution 4.0 International license.

**FIG 1:**
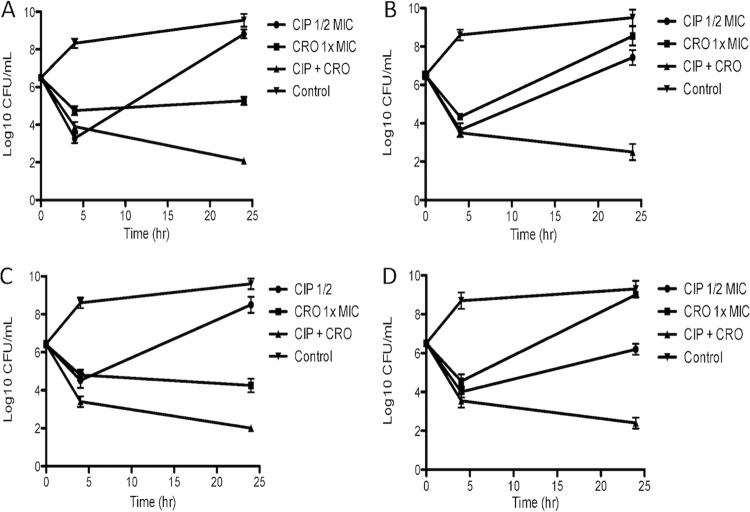
Representative killing assays combining 1× MIC of ceftriaxone (CRO) with 1/2 MIC of ciprofloxacin (CIP) for a *Salmonella* CSF isolate (MHB in panel A and RPMI+10% LB in panel B) and bloodstream isolate (MHB in panel C and RPMI+10% LB in panel D).

Checkerboard studies between CRO, CIP, and AZM are shown in Table 2, with synergy exhibited between (i) CRO and CIP in RPMI+10% LB, (ii) CRO and CIP in CA-MHB, and (iii) CRO and AZM in RPMI+10% LB based on fractional inhibitory concentration index (FICI). Additionally, time-kill assays using both standard bacteriologic CA-MHB and supplemented mammalian tissue culture medium RPMI+10% LB were performed to assess synergy and bactericidal activity for CIP+CRO, and the results are shown in Fig. 1. For both CSF and blood *Salmonella* isolates, the combination CRO+CIP in both CA-MHB and RPMI+10% LB demonstrated bactericidal activity (>3-log_10_-CFU/ml killing), whereas each drug alone resulted in either growth or stasis at the concentrations used (1× MIC of CRO, 1/2 MIC of CIP [Fig. 1]).

**TABLE 2:** FICIs of checkerboard assays between antibiotics using a *Salmonella* CSF isolate

Combination^a^	FICI in:	
	RPMI	MHB
CRO+CIP	0.3125	0.50
CRO+AZM	0.50	NA^b^
CIP+AZM	1	NA
CRO+LL-37	0.5	NA
CIP+LL-37	0.5	NA
AZM+LL-37	0.75	NA
CM+LL-37	1	NA

a CRO, ceftriaxone; CIP, ciprofloxacin; AZM, azithromycin; CM, chloramphenicol.

b NA, not applicable, as AZM and LL-37 are not active in MHB.

### Pharmacodynamic interaction between human cathelicidin LL-37 and administered antibiotics.

Both the CSF and bloodstream *Salmonella enterica* isolates showed an LL-37 MIC of 32 μM (Table 1).

**FIG 2:**
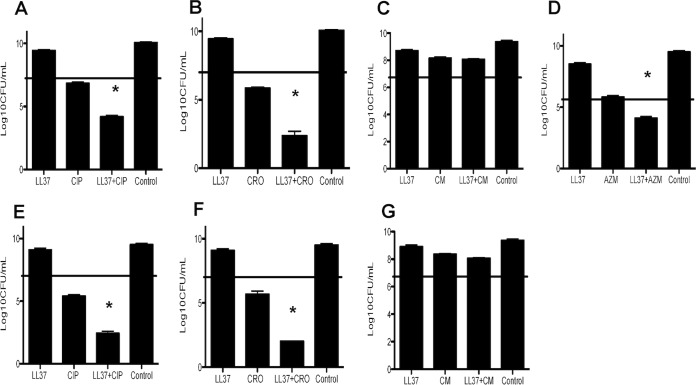
Assessment of activity of LL-37 (16 mM) in combination with various antibiotics against *Salmonella*. Shown are results of CFU counts of a *Salmonella enterica* CSF isolate (A, B, C, and D) or blood isolate (E, F, and G) in the presence of 1/2 MIC of LL-37 and 1/2 MIC of antibiotic after 6 h. The starting inoculum is shown by the horizontal line. CIP, ciprofloxacin; CRO, ceftriaxone; CM, chloramphenicol; AZM, azithromycin; Control, medium alone. *, *P* < 0.05 versus either agent alone.

The pharmacodynamic relationships between various antibiotics (CRO, CIP, AZM, and CM) and LL-37 were first determined by checkerboard assays. Results in Table 2 demonstrate synergy between CRO and LL-37 (FICI = 0.5) and CIP and LL-37 (FICI = 0.5) and additivity for AZM+LL-37 (FICI = 0.75) and CM+LL-37 (FICI = 1). Killing assays using 1/2 MIC of LL-37 (16 μM), with or without 1/2 MIC of CIP, CRO, AZM, and CM, were performed. The killing assay results in Fig. 2 demonstrate a 2- to 3-log_10_-CFU/ml reduction of viable bacteria at 6 h for LL-37+CIP, LL-37+CRO, and LL-37+AZM. However, there was no killing observed when combining LL-37 and CM.

**FIG 3:**
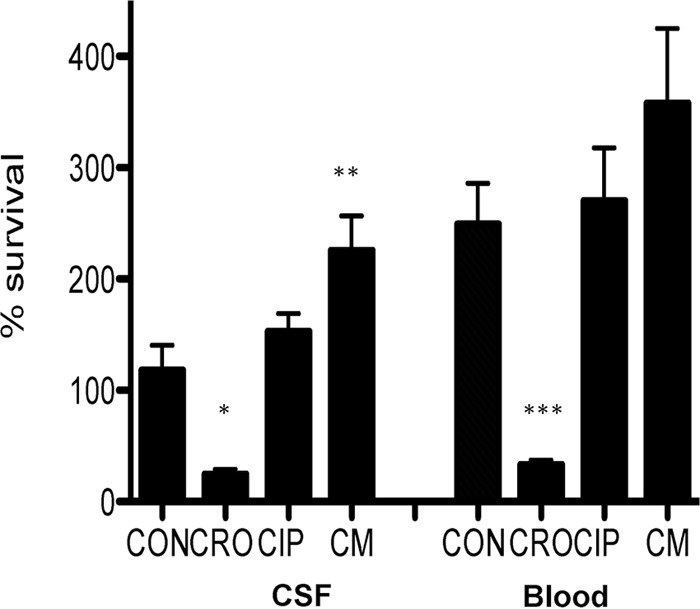
Assessment of activity of LL-37 (16 mM) against CSF and blood isolates from a patient with *Salmonella enterica* meningitis after growth in LB broth containing 0.05 mg/liter ceftriaxone (CRO), 0.0125 mg/liter ciprofloxacin (CIP), or 0.5 mg/liter chloramphenicol (CM) compared to antibiotic-free LB (control [CON]). *, *P* = 0.002 versus control; **, *P* = 0.04 versus control; ***, *P* = 0.006 versus control.

We next assessed the influence of overnight pretreatment of *Salmonella enterica* with sub-MICs of CRO, CIP, or CM on subsequent bacterial killing by LL-37. Figure 3 demonstrates that growth in sub-MIC CRO greatly sensitizes the pathogen to killing by LL-37 in both strains (*P* = 0.002 for the CSF isolate and *P* = 0.006 for the blood isolate versus the paired antibiotic-untreated control). However, this phenomenon was not observed for CIP or CM, which attenuated the killing activity of LL-37. This achieve significance only with chloramphenicol against the CSF strain (*P* = 0.04 versus control).

### Antibiotic exposure and sensitization to innate immune-mediated killing.

**FIG 4:**
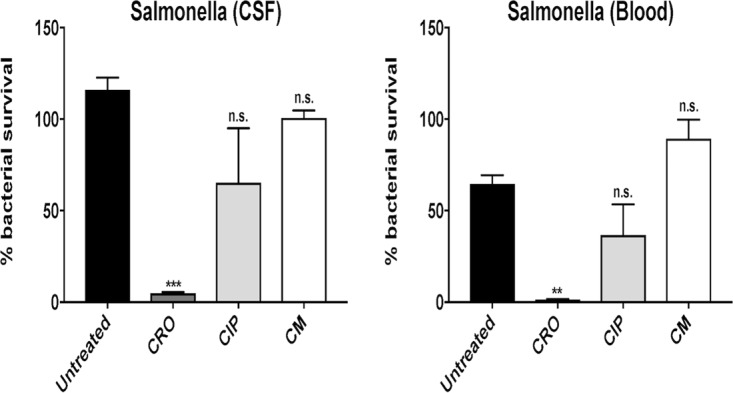
Neutrophil killing assays of CSF and blood *Salmonella* isolates were performed following exposure to antibiotic-free medium (untreated) or pretreatment with 1/4 MIC of ceftriaxone (CRO), 1/4 MIC of ciprofloxacin (CIP), or 1/4 MIC of chloramphenicol (CM). CRO sensitizes *Salmonella enterica* serotype Newport to neutrophil killing. Data are plotted as mean ± SEM and represent the percentage of bacterial survival remaining following 15 min of neutrophil exposure compared to the initial inoculum. **, *P* < 0.005, and n.s., no statistical significance, by two-way analysis of variance (ANOVA) and comparing pretreated bacteria to untreated bacteria.

**FIG 5:**
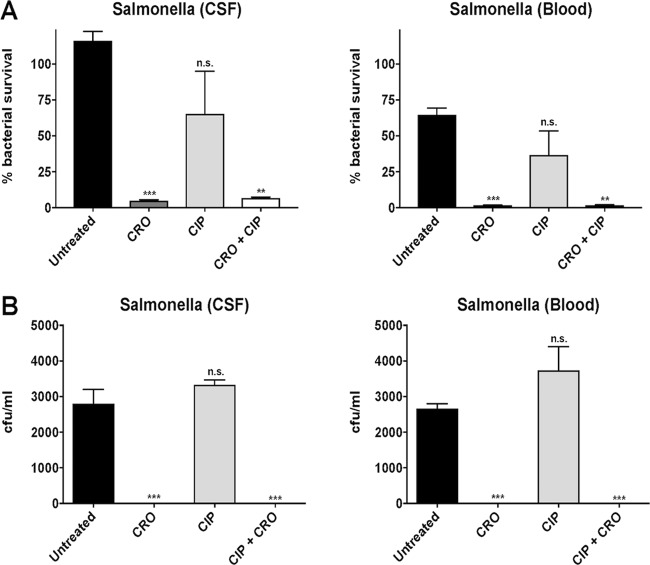
The percentage of bacterial survival and number of colony-forming units per milliliter of bacteria recovered from (A) neutrophil killing and (B) whole-blood killing assays, respectively, were significantly reduced for *Salmonella enterica* serotype Newport CSF and blood isolates pretreated with 1/4 MIC of ceftriaxone (CRO) and 1/4 MIC of CRO + 1/4 MIC of ciprofloxacin (CIP) but not 1/4 MIC of CIP alone. Data are plotted as mean ± SEM and represent the percentage of bacterial survival remaining following 15 min of neutrophil exposure compared to the initial inoculum or CFU per milliliter recovered following 60 min of exposure to whole blood. **, *P* < 0.005, ***, *P* < 0.0005, and n.s., no statistical significance, by two-way ANOVA and comparing pretreated bacteria to untreated bacteria.

**FIG 6:**
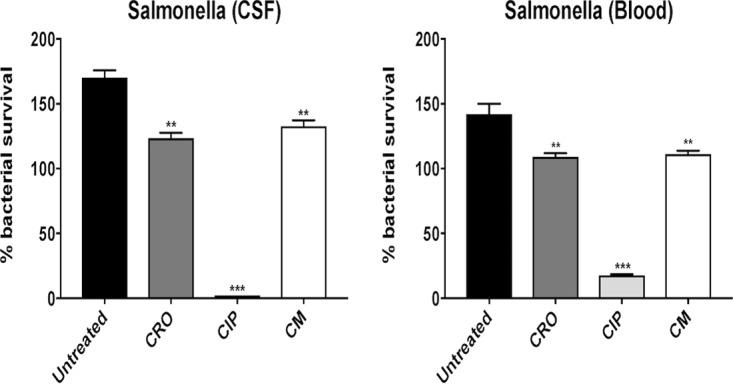
Neutrophil killing assays of CSF and blood *Salmonella* isolates pretreated with 1/4 MIC of LL-37 were performed following exposure to antibiotic-free medium (untreated) or 1× MIC of ceftriaxone (CRO), 1× MIC of ciprofloxacin (CIP), or 1× MIC of chloramphenicol (CM). Pretreatment with LL-37 sensitizes *Salmonella enterica* serotype Newport to neutrophil killing in the presence of 1× MIC of CRO, 1× MIC of CIP, and 1× MIC of CM. The percentage of bacterial survival was most prominently reduced in the presence of CIP. Data are plotted as mean ± SEM and represent the percentage of bacterial survival remaining following 15 min of neutrophil exposure compared to the initial inoculum. **, *P* < 0.005, ***, *P* < 0.0005, and n.s., no statistical significance, by two-way ANOVA and comparing bacteria with antibiotic to bacteria without antibiotic (untreated).

Neutrophil killing assays of CSF and blood *Salmonella* isolates were performed following exposure to antibiotic-free medium (untreated) or pretreatment with 1/4 MIC of CRO, 1/4 MIC of CIP, or 1/4 MIC of CM. CRO sensitizes *Salmonella enterica* serotype Newport to neutrophil killing, whereas CM and CIP do not (Fig. 4). Sub-MIC concentrations of CRO and CRO+CIP sensitize *Salmonella* to neutrophil killing (Fig. 5A) and whole-blood killing (Fig. 5B), whereas CIP alone does not. Additionally, neutrophil killing assays of the *Salmonella* isolates pretreated with 1/4 MIC LL-37 were performed following exposure to antibiotic-free medium (untreated) or 1× MIC of CRO, 1× MIC of CIP, or 1× MIC of CM (conditions predetermined by pilot studies). Pretreatment with LL-37 notably sensitized the patient’s *Salmonella enterica* serotype Newport isolates to neutrophil killing in the presence of 1× MIC of CRO, 1× MIC of CIP, and 1× MIC of CM (Fig. 6).

## DISCUSSION

Limitations of *in vitro* antimicrobial susceptibility testing methods as predictors of *in vivo* treatment efficacy are increasingly being recognized (12, 13). Such limitations in assays traditionally performed in bacterial growth media stem from two basic interrelated concepts: (i) the inability of bacterial media to replicate the *in vivo* environment (e.g., pH, availability of nutrients, and drug permeability) and (ii) the absence of host innate immunity factors that likely have important pharmacodynamic interactions with administered antibiotics. For example, we have previously shown the clinical importance of β-lactams as adjunctive therapy in treating β-lactam-resistant Gram-positive pathogens, such as methicillin-resistant *Staphylococcus aureus* (14, 15) and vancomycin-resistant *Enterococcus* (16), and that the macrolide antibiotic AZM may demonstrate excellent *in vivo* activity against some resistant Gram-negative bacteria (17, 18). A recent study of *Salmonella* that demonstrated tissue-specific selection of antimicrobial-resistant subpopulations that may serve as a reservoir of treatment failure that escapes detection by standard bacteriological media (9) provides further evidence that the paradigms of antimicrobial susceptibility testing established decades ago require a reevaluation for improvement.

While antimicrobials used to treat invasive S. enterica serovar Typhimurium infections have been examined extensively *in vitro* and in animal models of infection (19), the role of host immunity factors in enhancing their activities has not been examined. In this study, we found (i) consistent synergy between CRO and CIP (the agents used to successfully treat the patient) against both the CSF and blood isolates in physiologic RPMI+10% LB by checkerboard and time-kill assays, (ii) synergistic killing of *Salmonella* by combinations of sub-MICs of LL-37 and CRO, CIP, and AZM, with a most pronounced effect between CRO and LL-37, and (iii) sensitization to killing of *Salmonella* by human neutrophils and whole blood after growth in 1/4 MICs of CRO and CRO+CIP, but not CIP or CM. CM, a bacteriostatic agent against the *Salmonella* strains tested, showed no synergy with LL-37.

Due to its rarity, the treatment of *Salmonella* meningitis is far from standardized. In the largest assessment to date, Owusu-Ofori and Scheld describe 126 cases: most infections were due to *Salmonella* Typhimurium, and all but 2 occurred in children less than 2 years of age (4). In this retrospective analysis, clinical outcomes were significantly better in patients receiving 3rd-generation cephalosporins, quinolones, or imipenem compared to conventional therapies such as CM, clotrimoxazole, or ampicillin. Among the latter group, CM (the only bacteriostatic agent examined in this study) fared the most poorly in that 35 out of 54 patients (65%) relapsed or died (4). This compared to successful treatment in 83% and 89% of patients receiving 3rd-generation cephalosporins and quinolones, respectively (4). Paralleling this inferior clinical performance, the results from this study with CM showed (i) bacteriostatic activity at 2×, 4×, and 8× MIC in both RPMI+10% LB and CA-MHB media and (ii) additive activity with LL-37 (FICI = 1). This contrasted with CRO and CIP, which were each noted to have bactericidal activity at 2×, 4×, and 8× MIC in both RPMI+10% LB and CA-MHB media and synergy in combination with LL-37 (FICI = 0.5). While prior work has shown antagonism of LL-37 activity by bacteriostatic antibiotics against *Escherichia coli* and *S. aureus* (20), the lack of synergy of CM with LL-37 demonstrates another collateral benefit of bactericidal antibiotics in that they enhance bacterial killing by innate immune mechanisms. These data highlight the importance of antimicrobial activity beyond the “susceptible versus resistant” designation reported by clinical microbiology laboratories when treating serious infections such as meningitis.

The effects of various antibiotics on the sensitivity of *Salmonella* to killing by components of human innate immunity were of interest. Cathelicidin LL-37 is a critical host defense antimicrobial peptide in the pathogenesis of bacterial meningitis, and therefore cooperative activity between LL-37 and the antibiotics used to treat meningitis is anticipated to be an important parameter to ensure successful treatment (10). While this has not been extensively studied, we previously found that ceftaroline demonstrated this synergy with LL-37 to a much greater degree than vancomycin, paralleling its superior performance in a difficult case of *Streptococcus pneumoniae* meningitis (11). In the present study, we found that pretreatment of the *Salmonella* isolates with 1/2 MIC of CRO, CIP, or AZM enhanced killing by LL-37 but resulted in either stasis or growth alone (Fig. 2).

It is worth mentioning that AZM has been used successfully to treat enteric fever due to *Salmonella* (21). While the relationship of MICs measured in standard microbiological media such as CA-MHB to clinical outcome is not entirely clear, the demonstration of a severalfold reduction in AZM MIC in RPMI+10% LB medium suggests that further clinical-microbiological correlation studies with AZM are warranted. A recent study demonstrated that the addition of bicarbonate to standard bacteriological media approximates susceptibility testing results obtained in physiologic media (13). The same investigators demonstrated that the susceptibility results obtained using bicarbonate-buffered bacteriological media or physiologic media were more reflective of antimicrobial performance *in vivo* using animal models of infection (13). It appears the *in vivo* activity of AZM against *Salmonella* may be vastly underappreciated using conventional antimicrobial susceptibility methods, a theme that appears to be recurring with Gram-negative bacteria given previously published data (17, 18, 22). A more accurate assessment of antibiotic activity *in vivo* may be appreciated by considering antibiotic interactions with various components of the innate immune system, understanding variability in antibiotic activity based on bacterial inoculum, and utilizing susceptibility testing conditions that better recapitulate the host environment, including medium type and pH.

## MATERIALS AND METHODS

### Bacterial strains.

The *Salmonella enterica* serotype Newport strains examined in this study were the bloodstream and cerebrospinal fluid (CSF) isolates from a patient who was successfully treated and whose lumboperitoneal shunt was salvaged without removal using CRO and CIP.

### Antimicrobial susceptibility testing.

CIP, CRO, and CM were purchased from Sigma-Aldrich (St. Louis, MO). AZM in 500-mg lyophilized vials was purchased from Baxter Healthcare (Deerfield, IL). Human cathelicidin LL-37 was purchased from AnaSpec, Inc. (Fremont, CA), diluted to a stock concentration of 640 μM, and stored as frozen single-use 50-μl aliquots. Antimicrobial susceptibility testing and checkerboard assays were performed in cation-adjusted Mueller-Hinton broth (CA-MHB) and in RPMI 1640 medium supplemented with 10% Luria-Bertani broth (RPMI+10% LB) according to CLSI methods using freshly thawed bacteria from the −80°C freezer plated on Todd-Hewitt agar (THA) (23). Checkerboard assays (8 by 8) were run twice, and the results were interpreted by calculating the fractional inhibitory concentration index (FICI). The FICI is calculated using the formula FICabx_1_ + FICabx_2_ = FICI, where FICabx_1_ = the MIC of antibiotic 1 in combination divided by the MIC of antibiotic 1 alone and FICabx_2_ = the MIC of antibiotic 2 in combination divided by the MIC of antibiotic 2 alone. An FICI of <0.5 defines synergy, >0.5 to <1.0 additivity, >1 to <4.0 indifference, and >4.0 antagonism (24). *In vitro* killing assays were performed with single drugs and in various combinations using CA-MHB or RPMI+10% LB with starting inocula of approximately 5 × 10^6^ CFU/ml, and sampling was performed at 4 and 24 h. LL-37 killing assays were performed in RPMI+10% LB using starting inocula of 5 × 10^5^ CFU/ml as previously described. Bacterial survival was assessed at 2 h in the LL-37 killing assays on antibiotic-exposed bacteria and at 6 h in the studies involving LL-37 combined with antibiotics. For the experiments examining the effects of antibiotics on sensitization to LL-37 killing, bacteria were grown overnight in CA-MHB, pelleted, washed once in phosphate-buffered saline (PBS), and then resuspended in fresh RPMI+10% LB for addition to LL-37 killing assays. Bacterial counts were enumerated on THA plates.

### Neutrophil killing assays.

Human neutrophils were isolated from healthy donors using the PolymorphPrep system (Axis-Shield) under protocols approved by the University of California San Diego Human Subjects Institutional Review Board for use in established bacterial killing assays with minor modifications (14). Neutrophils were used to seed a 96-well plate (2 × 10^5^ cells/well). In experiment A, cells were infected at a multiplicity of infection (MOI) of 10 with *Salmonella* CSF or blood isolates that were grown overnight without antibiotic or with antibiotics (1/4 MIC of CRO, 1/4 MIC of CIP, 1/4 MIC of CM, or 1/4 MIC CRO + 1/4 MIC of CIP). In experiment B, cells were infected at a multiplicity of infection (MOI) of 10 with *Salmonella* CSF or blood isolates grown to stationary phase overnight with 1/4 MIC of LL-37 and then exposed to neutrophils without antibiotics or with antibiotics (1× MIC of CRO, 1× MIC of CIP, or 1× MIC of CM). After incubation for 15 min at 37°C and 5% CO_2_, the cells were lysed with 0.025% Triton X-100 and serially diluted in phosphate-buffered saline (PBS), and the total number of remaining bacteria was enumerated on Luria agar (LA) plates. The percentage of bacterial survival at 15 min was calculated as the mean ± standard error of the mean (SEM) of the initial inoculum.

### Whole-blood killing assay.

Blood from healthy donors was collected using heparinized syringes. *Salmonella* CSF or blood isolates were grown to stationary phase overnight without antibiotic or with antibiotic (1/4 MIC of CRO, 1/4 MIC of CIP, 1/4 MIC of CM, or 1/4 MIC of CRO + 1/4 MIC of CIP). A total of 2 × 10^3^ CFU of the stationary-phase bacteria were mixed with 200 μl of whole human blood in siliconized tubes and incubated at 37°C with orbital rotation for 60 min, 25-µl aliquots were removed, and cells were lysed with 0.025% Triton X-100, serially diluted in PBS, and plated onto LA for enumeration of surviving CFU (25, 26). These studies were approved by the University of California San Diego Human Research Protections Program.
