# Supplementary material for: Interaction of Antibiotics with Innate Host Defense Factors against Salmonella enterica Serotype Newport
Source: mSphere. 2017 Dec 6;2(6):e00410-17. doi: 10.1128/mSphere.00410-17 (PMC5717323; doi:10.1128/mSphere.00410-17)
Supplement: FIG S1 [file sph006172421sf1.pdf]

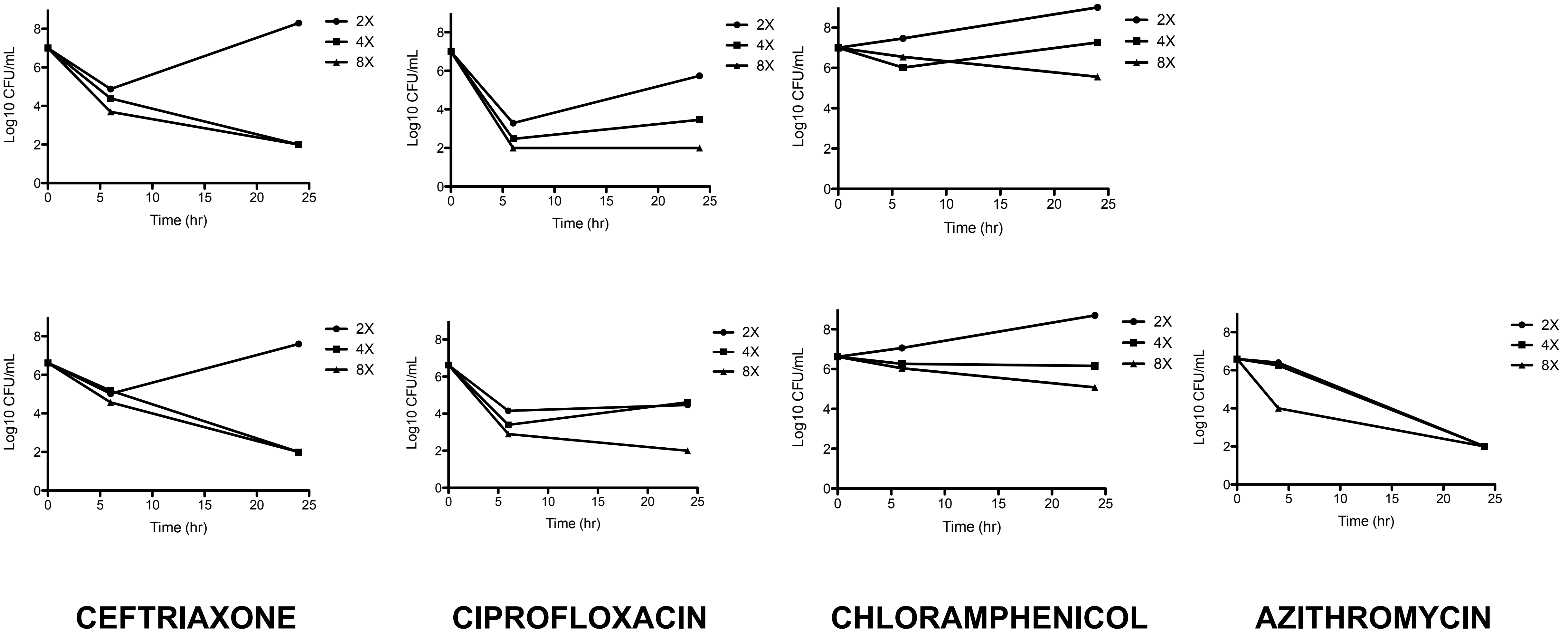

Figure S1. Kill curves against CSF Salmonella enterica strain in MHB (top row) and RPMI+10%LB (bottom row) using various antibiotics at 2X, 4X, and 8X MIC. Azithromycin was assayed only in RPMI+10%LB as it was inactive in MHB.
